# Hypocapnia as a poor prognostic factor in aneurysmal subarachnoid hemorrhage

**DOI:** 10.1186/2045-9912-3-25

**Published:** 2013-11-25

**Authors:** Paul Foreman, Christoph J Griessenauer, Mohammadali M Shoja, R Shane Tubbs

**Affiliations:** 1 Department of Neurosurgery, Univeristy of Alabama at Birmingham, Birmingham, AL, USA; 2 Pediatric Neurosurgery, Children’s Hospital, Birmingham, AL, USA

**Keywords:** Hypocapnia, CO_2_, Cerebral vasospasm, Subarachnoid hemorrhage, Outcome

## Abstract

In this editorial, the issues of hypocapnia and its relation to symptomatic vasospasm, prognosis, and outcome among patients with aneurysmal subarachnoid hemorrhage is discussed. Potential directions for future studies are provided.

## 

Global incidence of aneurysmal subarachnoid hemorrhage (aSAH) is estimated at approximately 10 per 100,000, with some 20,000-30,000 new cases occurring each year in the United States. Despite comprising only 5% of strokes, aSAH accounts for a significant proportion of stroke-related morbidity and mortality. In-hospital mortality rate approaches nearly 30% and approximately 10-20% of survivors remain functionally dependent. Despite overall improvements in fatality and outcome in recent decades, aSAH still remains a significant source of stroke-related morbidity and mortality. This has led to an intensive research effort to improve the care and outcome of these critically ill patients [1].

The impact of hyperventilation with subsequent hypocapnia in the setting of traumatic brain injury (TBI) has been studied extensively. Carbon dioxide (CO_2_) is a potent dilator of the cerebrovasculature and induced hyperventilation is an established mean to reduce the intracranial pressure (ICP) through cerebral vasoconstriction. While effective in reducing elevated ICP, the vasoconstrictive effect of hypocapnia can exacerbate cerebral ischemia [2] in the traumatized brain producing deleterious effects on the neurologic outcome [3]. In addition to the deleterious effects on the CNS, hypocapnia has also been shown to negatively impact organ-tissue perfusion of subcutaneous tissue and muscle [4], stomach [5,6], and bowel [7]. This has led to guideline recommendations against the use of prophylactic hyperventilation in the setting of TBI; rather, hyperventilation is only to be used as a temporizing measure for the reduction of elevated ICP [8]. Similar deleterious findings have been demonstrated in the setting of acute cerebrovascular accidents (CVA) [9]. An early study evaluating the association between respiratory patterns and arterial blood gas tensions in the setting of CVA found hyperventilation, hypocapnia, and arterial alkalosis to be associated with poor prognosis [9]. While much of the current literature focuses on the effects of permissive hyperventilation, a recent prospective study of spontaneous hyperventilation produced similar results [10].

Cerebral blood flow is compromised following aSAH [11]. While permissive hyperventilation is rarely utilized for the treatment of elevated ICP in aSAH patients, the development of spontaneous, central hyperventilation with resultant hypocapnia has been observed in aSAH patients. Concerns about the effect of this spontaneous reduction of CO_2_ on the cerebral blood flow in these patients has led to investigation into the incidence of hypocapnia and its relationship with outcome in aSAH patients [12]. Solaiman et al. have retrospectively assessed a cohort of 102 mechanically ventilated patients suffering an aSAH in an effort to demonstrate the incidence of hyperventilation and hypocapnia, as well as to determine its association with symptomatic vasospasm and clinical outcome [12]. Hypocapnia, as defined by a PaCO_2_ of less than 35 mmHg, was reported at least once during the intensive care unit stay in 99% of patients. Ninety-four patients (92%) had at least 1 day in which all PaCO2 measurements were below 35 mm Hg. Sixty-three percent of patients had episodes with PaCO_2_ of less than 30 mm Hg. Median duration of hypocapnia (defined by the number of days in which any PaCO2 measurement in a given day was <35 mm Hg) was 4 days. Interestingly, the majority of patients were spontaneously breathing with minimal ventilator support suggesting a central mechanism for hyperventilation. Intentional hyperventilation for impending herniation was not performed. The duration of hypocapnia was found to be independently associated with symptomatic vasospasm [adjusted odds ratio (OR) = 1.25 (95% CI, 1.07-1.47)], and unfavorable outcome [adjusted OR = 1.33 (95% confidence interval (CI), 1.04-1.70)]. Despite the statistically significant association, it should be noted that this remains an association rather than proof of causation. Regardless, given what is known about hypocapnia and cerebral blood flow, it makes sense that a reduction of CO_2_ could lead to reduced cerebral blood flow [10,13,14], exacerbation of cerebral ischemia [2], and an increase in extracellular mediators of secondary brain injury [13]. It is very likely that the added physiologic stress from hypocapnia-induced brain ischemia, serves to exacerbate the already reduced cerebral blood flow present following aSAH, placing the patient at increased risk for cerebral infarction.

The identification of hypocapnia as a poor prognostic factor among aSAH patients raises the question as to whether tight PaCO_2_ control would improve outcome following aSAH or not [13]. This required ventilatory control through sedation and neuromuscular blockade, which by itself diminishes the quality of a clinical neurologic assessment and places the patients at risk for unidentified neurologic decline. While other modes of assessment (i.e. ICP monitoring, transcranial Doppler) can be simultaneously performed, they lack the sensitivity of a skilled neurologic exam. Therefore, aggressive control of a patient’s ventiliatory status in exchange for the clinical neurologic exam may not be advisable. However, it can be assumed that avoidance of induced hyperventilation (in the absence of impending herniation) and the use of moderate sedation to target normocapnia is a reasonable approach. Based on the available data, it seems that further studies would be useful to fully elucidate the role of CO_2_ levels and its potential therapeutic and prognostic implications in the setting of aSAH. Whether or not a tight PaCO2 control would improve the outcome in this setting requires careful consideration and should be addressed in clinical trials.

## Competing interests

The authors declare that they have no competing interest.
